# In this issue of the *Korean Journal of Women Health Nursing* June 2020

**DOI:** 10.4069/kjwhn.2020.06.24

**Published:** 2020-06-24

**Authors:** Sue Kim

**Affiliations:** College of Nursing, Yonsei University, Seoul, Korea

We are pleased to share the June 2020 issue of the *Korean Journal of Women Health Nursing*.

Our issue article on COVID-19 provides a succinct and up-to-date briefing on the impact of the pandemic on women’s health, especially within the Korean context.

This issue also includes two review articles and several original studies on the following topic clusters:

**• *Review articles:*** A scoping review on prenatal nursing interventions conducted in Korea, covering the past four decades. Also a literature review of studies on women’s health promotion over the past 25 years, that applied Cox’s Interaction Model of Client Health Behavior (1982) as theoretical framework.

**• *Reproductive health:*** A secondary analysis study reports on early menopause status and differences in health-related quality of life. Another study reports on an educational intervention for women with benign uterine tumors receiving high-intensity focused ultrasound, which is a relatively new treatment modality that is being increasingly used. A study on development of a needs assessment scale for nursing care in women with infertility is also presented. Another study reports on premenstrual syndrome in nurses, specifically in relation to exposure to endocrine disruptors, emotional labor, and peer support.

**• *Maternal health:*** A study investigates how couples’ attitudes on sex during pregnancy affect sexual function. Also an intervention study for high-risk pregnant women in the maternal-fetal intensive care unit reports its impact on uncertainty, anxiety, and maternal-fetal attachment. Another study examines sleep quality of women with preterm labor and how it is affected by anxiety and smartphone dependency.

We trust readers will enjoy the diverse topics in this issue and hope everyone will stay safe and keep well.

